# Chemotactic preferences govern competition and pattern formation in simulated two-strain microbial communities

**DOI:** 10.3389/fmicb.2015.00040

**Published:** 2015-02-02

**Authors:** Florian Centler, Martin Thullner

**Affiliations:** Department of Environmental Microbiology, UFZ – Helmholtz Centre for Environmental ResearchLeipzig, Germany

**Keywords:** chemotaxis, pattern formation, competition, coexistence, communities, individual-based modeling

## Abstract

Substrate competition is a common mode of microbial interaction in natural environments. While growth properties play an important and well-studied role in competition, we here focus on the influence of motility. In a simulated two-strain community populating a homogeneous two-dimensional environment, strains competed for a common substrate and only differed in their chemotactic preference, either responding more sensitively to a chemoattractant excreted by themselves or responding more sensitively to substrate. Starting from homogeneous distributions, three possible behaviors were observed depending on the competitors' chemotactic preferences: (i) distributions remained homogeneous, (ii) patterns formed but dissolved at a later time point, resulting in a shifted community composition, and (iii) patterns emerged and led to the extinction of one strain. When patterns formed, the more aggregating strain populated the core of microbial aggregates where starving conditions prevailed, while the less aggregating strain populated the more productive zones at the fringe or outside aggregates, leading to a competitive advantage of the less aggregating strain. The presence of a competitor was found to modulate a strain's behavior, either suppressing or promoting aggregate formation. This observation provides a potential mechanism by which an aggregated lifestyle might evolve even if it is initially disadvantageous. Adverse effects can be avoided as a competitor hinders aggregate formation by a strain which has just acquired this ability. The presented results highlight both, the importance of microbial motility for competition and pattern formation, and the importance of the temporal evolution, or history, of microbial communities when trying to explain an observed distribution.

## Introduction

Microbial life populating natural habitats such as soils is usually found to be abundant and of astonishingly high diversity. The fate of an individual cell is hence not only determined by its abiotic environment but also by the presence of cells which might share its metabolic profile, or differ significantly. As a result, a multitude of ecological interactions is thought to shape microbial communities (Hibbing et al., 2010; Faust and Raes, 2012) and with it the essential ecosystem services they provide, from the local recycling of nutrients to global elemental cycles. The analysis of microbial co-occurrence patterns has provided evidence that—opposed to cooperation—competition between co-occurring microbes is the more common mode of interaction in both, natural environments (Chaffron et al., 2010) and the human gut microbiome (Levy and Borenstein, 2013). Laboratory experiments with bacterial strains isolated from aquatic environments support this hypothesis (Foster and Bell, 2012). Hence, microbial species of similar metabolic capacity often share a habitat and compete for common resources including nutrients and space.

While growth kinetics are clearly an important factor in competition, other physiological traits such as motility can play an equally important role. For example, it was shown in a theoretical study that in the presence of nutrient gradients an inferior growth phenotype can be compensated for by a superior chemotactic response (Kelly et al., 1988). Chemotaxis is the ability of microbial cells to detect chemical gradients in their environment and to align their flagella or pili mediated locomotion along such gradients. This capacity allows them to seek out locations providing favorable growth conditions and to avoid toxic environments. A large number of compounds has been identified that act as chemoattractants including sugars, amino acids, oxygen, nucleotides, and vitamins (for a comprehensive list relevant to *Pseudomonas* see Sampedro et al., 2014). Chemotaxis influences microbial transport in saturated porous media (Ford and Harvey, 2007) and can enhance bioremediation as many pollutants act as chemoattractants (Marx and Aitken, 2000; Pandey and Jain, 2002). Besides responding to chemical gradients imprinted on their environment by abiotic factors such as preferential flow paths, microbial cells have also been reported to react chemotactically to compounds which are secreted by the cells themselves. This behavior enables the formation of bacterial aggregates (Mittal et al., 2003; Park et al., 2003) and might offer survival benefits. Similar to biofilms, an aggregated lifestyle might provide advantages during fluctuating environmental conditions and protects against predation (Hahn et al., 2000; Corno and Jurgens, 2008). Enhanced resistance to antibiotics has been reported (Butler et al., 2010), and antibiotics can even induce co-aggregation (Corno et al., 2014). Furthermore, close physical proximity allows for the efficient utilization of excreted products including extracellular enzymes that break down complex molecules for microbial uptake (Folse and Allison, 2012) and siderophores for iron scavenging (Kümmerli et al., 2014).

Microbial systems featuring chemotactic microbes have extensively been modeled mathematically (Tindall et al., 2008). However, only few studies considered the case that bacteria respond to more than one chemoattractant. Diverse spatio-temporal patterns from homogeneous distributions and inhomogeneous static patterns to traveling waves were observed if bacteria are assumed to respond simultaneously to substrate and a self-excreted compound as two chemoattractants (Saragosti et al., 2010; Centler et al., 2011; Curk et al., 2013; Gharasoo et al., 2014). We consider such a case in this theoretical study and explore its consequences for competition in a two-species community. While the relation between chemotaxis and growth (Kelly et al., 1988; Yan et al., 2014), and trade-offs between growth rate and yield, or growth rate and substrate affinity have been considered before (Gudelj et al., 2007), this study focuses on a trade-off regarding motility only. Cells of two motile bacterial strains sharing the same growth phenotype (i.e., identical maximum specific growth rates, yields and substrate affinities) compete in a two-dimensional environment for a common substrate. Strains only differ in their chemotactic preference, either being more attracted by the self-excreted chemoattractant or by the substrate. The complexity of chemotactic pathways (Porter et al., 2011) makes the existence of strains featuring a broad range of chemotactic responses plausible. At the extreme ends of the chemotactic preference range, strains follow an aggregated or a highly motile lifestyle, comparable to behavioral strategies which have been reported for recently speciated populations of marine bacterioplankton sharing similar growth and chemotactic capabilities. While one species attaches itself to nutrient-rich particles and forms biofilms, the other remains motile and hence can respond more rapidly to the arrival of new nutrient particles (Yawata et al., 2014).

While abiotic heterogeneities certainly modulate microbial interactions (Vos et al., 2013; Gharasoo et al., 2014), we focus in this study on a homogeneous environment, so that observations can be directly attributed to microbial properties. The aim of this study is to analyze the impact of chemotactic preference as a microbial motility phenotype on competition in a simulated two-strain community. In the absence of growth advantages and abiotic heterogeneities: How does the chemotactic preference influence community fate and spatio-temporal distribution patterns, and what is an optimal strategy?

## Materials and methods

### Model description

An established model developed for microbial growth and motility in a one-dimensional domain (Centler et al., 2011) and subsequently expanded to two dimensions (Gharasoo et al., 2014) has been adapted for the present study.

Microbial cells' biomass is assumed to grow in dependence of substrate availability following Monod kinetics with an additional term for maintenance:

(1) $$\frac{dX}{dt}=\mu \frac{S}{K_{M}+S}X-mX,$$

with *X* referring to the cells' dry biomass [g], maximum specific growth rate μ [1/h], substrate concentration *S* [g/l], Monod constant *K_M_* [g/l], and maintenance rate term *m* [1/h]. Substrate is supplied to the domain by a generic release process (e.g., dissolution) and consumed by bacteria:

(2) $$\frac{dS}{dt}=\lambda \left(S_{in}-S\right)-\mu \frac{S}{K_{M}+S}X\frac{1}{Y\cdot V},$$

with additional parameters λ [1/h] characterizing the substrate supply rate, maximum concentration of substrate in the aqueous phase *S_in_* [g/l], yield factor *Y* [g dry biomass/g substrate] and habitat volume *V* [l]. The non-trivial steady state at which biomass and substrate become constant are obtained by setting Equations (1) and (2) to zero:

(3) $$X^{*}=\lambda \cdot Y\cdot V\cdot \frac{K_{M}\cdot m+S_{in}\left(m-\mu \right)}{m\left(m-\mu \right)},\text{ type="pmid" type="pmid" type="pmid" }S^{*}=\frac{K_{M}\cdot m}{\mu -m}.$$

As described previously (Centler et al., 2011; Gharasoo et al., 2014), bacterial motility is assumed to be driven by three processes. First, an active, undirected movement of the bacteria is modeled as Fickian diffusion with a diffusive bacterial flux of *J_D_* = −*D* · ∇*p*, with bacterial diffusion coefficient *D* and number of bacterial cells *p*. Second, bacteria respond to gradients in substrate concentration and direct their movement toward higher concentrations. This is implemented in the model by the chemotactic flux *J_s_* = χ_*s*_ · *p* · ∇*S*, in which χ_*s*_ defines the chemotactic sensitivity of the bacteria with respect to substrate gradients. Thirdly, bacteria respond to chemicals which are emitted by themselves. For simplicity, these emitted chemicals are not modeled explicitly, and instead this behavior is implemented as a chemotactic flux which depends directly on the bacterial population gradient with *J_p_* = χ ·*p* · ∇*p*, with chemotactic sensitivity parameter χ. Note that when later two bacterial strains are considered, this flux will depend on the total population as excreted chemoattractants are assumed to be common amino acids such as aspartate or glycine which are produced and sensed by both strains. Substrate is supplied throughout the full domain homogeneously (i.e., λ and *S_in_* are constant in space) and is assumed to diffuse freely in the domain with diffusion coefficient *D_s_*. The full model is then described by two partial differential equations:

(4) $$\frac{\partial p}{\partial t}=D\cdot \nabla^{2}p-\chi_{s}\cdot \nabla \cdot \left(p\nabla S\right)-\chi \cdot \nabla \cdot \left(p\nabla p\right)+p_{local},$$

(5) $$\frac{\partial S}{\partial t}=D_{S}\cdot \nabla^{2}S+S_{local},$$

with *p_local_* and *s_local_* referring to the dynamics as defined by the growth model in Equations (1) and (2), respectively. Bacterial aggregation triggered by chemotaxis to bacteria can lead to unrealistic large bacterial densities. To avoid this, cells may not invade locations where bacterial density has exceeded a threshold *X_thr_*.

Different microbial strains are considered which share identical growth characteristics and differ only in their chemotactic behavior. Hence, only the chemotactic sensitivities to substrate and bacteria are modified to define a novel strain in the model. We assume that the ability to respond more sensitively to one attractant comes at the expense of sensitivity toward the other, implying a trade-off. To make one strain not more motile than the other we keep the total chemotactic flux of bacteria constant for all strains. (One strain being more motile than the other would imply that it directs more energy toward motility at the expense of growth, violating our assumption of identical growth phenotypes.) For a reference set of chemotactic sensitivities χ*^ref^_s_* and χ*^ref^*, the total chemotactic flux is given by *p* · (χ*^ref^_s_* · ∇*s* + χ*^ref^* · ∇*p*). For given representative gradients in bacteria ∇*p^r^* and substrate ∇*s^r^*, this flux is kept constant if for a desired chemotactic sensitivity to bacteria χ, the chemotactic sensitivity to substrate is chosen according to:

(6) $$\chi_{s}=\chi_{s}^{ref}+\left(\chi^{ref}-\chi \right)\cdot \frac{\nabla p^{r}}{\nabla s^{r}}.$$

We select typical values as reference values with χ*^ref^_s_* = 1.3 × 10^−3^ cm^2^/s (Berg and Turner, 1990) and χ*^ref^* = 5.0 × 10^−9^ cm^2^/s (Centler et al., 2011). As representative gradients are difficult to define, we instead select desired end points in which one chemotactic sensitivity is set to zero and the other is set to the two-fold reference value, or vice versa, leading to ∇*p^r^*/∇*s^r^* = 260. In this way we define strains that cover a broad range of chemotactic preferences from responding solely to substrate gradients (χ*_s_* = 2.6 × 10^−3^ cm^2^/s, χ = 0 cm^2^/s), to responding solely to bacterial gradients (χ*_s_* = 0 cm^2^/s, χ = 1.0 × 10^−8^ cm^2^/s). We identify the chemotactic preference of a strain by referring to its chemotactic sensitivity to bacteria, which implies a corresponding unique chemotactic sensitivity to substrate via Equation (6). By convention, the first strain of the community is always assigned with the higher affinity to bacteria and is referred to as the more aggregating strain.

### Individual-based model implementation

Space is discretized into a two-dimensional regular grid with a spacing of Δ*x* in both directions. Periodic boundary conditions are employed, such that the spatial simulation domain resembles a torus. Each grid location represents a habitat of volume Δ*x*^3^ and is available for colonization by bacterial cells. An operator splitting approach is used in which in each time step of length Δ*t* first, bacterial growth is simulated followed by the simulation of bacterial motility and substrate diffusion. For microbial growth, Equations (1) and (2) are solved using the fourth-order Runge-Kutta method at each spatial location separately. Microbial cells are individually characterized by their biomass *X_i_* where *i* identifies a particular cell. Instead of solving one differential equation for each individual cell, it is sufficient to solve Equation (1) defining the fate of the total population *X* as all cells share identical growth kinetics and the fate of an individual cell is linked to the fate of the total population via $\frac{dX_{i}}{dt}=\frac{dX}{dt}\cdot \frac{X_{i}}{X}$. If the total biomass changes from time *t* to *t*+Δ*t* by Δ *X^t^*, the biomass of cell *i* is hence changing by $\Delta X^{t}\cdot \frac{X_{i}(t)}{X(t)}$.

Bacterial cells are dividing if their biomass exceeds a biomass threshold *X_max_*. On cell division, the original cell is replaced by two daughter cells of the same strain (having identical parameter values for chemotaxis). While the sum of both daughter cells equals the biomass of the dividing cell, cell division is not fully symmetric. The deviation from a symmetric division is randomly drawn from a normal distribution with standard deviation *X_max_*/10. If cell biomass decreases below a threshold *X_min_*, set to a value slightly below *X_max_*/2, the cell dies and is removed from the simulation.

Microbial motility is governed by a stochastic approach in which probabilities to remain at a location or move to one of the eight neighboring locations are calculated based on Equation (4) as described previously (Gharasoo et al., 2014). After calculating the movement probabilities at all locations, cells are moved between locations according to these probabilities. If the movement of a cell would lead the biomass in the target location to exceed the threshold *X_thr_*, the cell is not transferred but remains in its current location. To avoid priority effects, at each time step a random location sequence is selected according to which cell migration is performed. As the last step, substrate diffusion is simulated by employing the standard central difference scheme.

Parameter values for microbial growth were chosen to reflect a fast growing microbial species such as *Escherichia coli* growing on glucose (Kreft et al., 1998). All model parameters are summarized in Table 1.

**Table 1:** **Model parameters**.

**Parameter**		**Value**	**Unit**	**Reference/Remarks**
Maximum specific growth rate	μ	1.23	1/h	Kreft et al., 1998
Monod constant	*K_m_*	2.34 × 10^−3^	g/l	Kreft et al., 1998
Yield factor	*Y*	0.4444	g dry biomass/g substrate	Kreft et al., 1998
Maintenance	*m*	0.016	1/h	Kreft et al., 1998
Substrate inflow rate parameter	λ	216	1/h	
Maximum substrate concentration	*S_in_*	3.0 × 10^−4^	g/l	chosen to be approx. 10 × S*
Spatial discretization	Δ*x*	5 × 10^−3^	cm	
Habitat volume	*V*	1.25 × 10^−10^	l	
Simulation domain size		0.5 × 0.5	mm × mm	1 × 1 for selected scenarios
Temporal discretization	Δ*t*	0.1	s	
Biomass at cell division	*X_max_*	1.45 × 10^−13^	g dry biomass	Kreft et al., 1998
Minimum cell biomass	*X_min_*	0.7 × 10^−13^	g dry biomass	
Substrate diffusion coefficient	*D_s_*	9 × 10^−6^	cm^2^/s	Berg, 1983
Bacterial diffusion coefficient	*D*	5.19 × 10^−6^	cm^2^/s	Berg and Turner, 1990
Chemotactic sensitivity toward substrate (reference)	*χ^ref^_s_*	1.3 × 10^−3^	cm^2^/s	Berg and Turner, 1990
Chemotactic sensitivity toward bacteria (reference)	*χ^ref^*	5.0 × 10^−9^	cm^2^/s	Centler et al., 2011
Range of chemotactic preference	(*χ_s_, χ*)	(2.6 × 10^−3^, 0)−(0, 1.0 × 10^−8^)	(cm^2^/s, cm^2^/s)	
Threshold for bacterial immigration	*X_thr_*	1.0875 × 10^−9^	g dry biomass	(Approximately 10.000 cells)

### Pattern formation threshold in mixed bacterial populations

A mathematical analysis of the spatial model in one dimension revealed that for spatial patterns to emerge, the chemotactic sensitivity to bacteria χ had to exceed a threshold χ*^T^* given by the quotient of the bacterial diffusion coefficient *D* and the steady state number of cells p^*^ in a habitat (Centler et al., 2011):

(7) $$\chi^{T}=\frac{D}{p^{*}}.$$

Two-dimensional model runs revealed that this condition is only mandatory but not sufficient for pattern formation as both patterns and homogeneous distributions were observed above this threshold (Gharasoo et al., 2014).

For a bacterial population consisting of two strains only differing in chemotactic sensitivities, this condition can be expanded as follows. If the first strain always features a higher (or equal) chemotactic affinity toward bacteria than the second (χ_1_ ≥ χ_2_), the share of the more aggregating strain on the total population is then given by:

(8) $$f:=\frac{p_{1}}{p_{1}+p_{2}},$$

with *p*_1_ and *p*_2_ referring to the number of cells of the first and second strain. We select an initial population composition *f*_0_ and initialize the model with a homogenous distribution of both strains, ensuring that the model is at steady state in each location (*p*(*x*, *y*) = p^*^ = *p*_1_+ *p*_2_, *S* (*x*, *y*) = S^*^). If the second strain shares the chemotactic preference of the first (χ_2_ = χ_1_), the situation resembles the situation of a monoculture. In this case, the pattern formation condition Equation (7) applies. If the second strain is not chemotactically attracted to bacteria at all (χ_2_ = 0), the second strain cannot contribute toward the tendency to form patterns, leaving only the first strain to initiate pattern formation. However, its cell count at steady state is smaller than p^*^ with p^*^_1_ = *f*_0_ · p^*^. This leads to the pattern formation threshold for the first strain which also depends on the initial community composition *f*_0_:

(9) $$\chi_{1}^{T}=\frac{D}{f_{0}\cdot p^{*}}.$$

**Figure 1:**
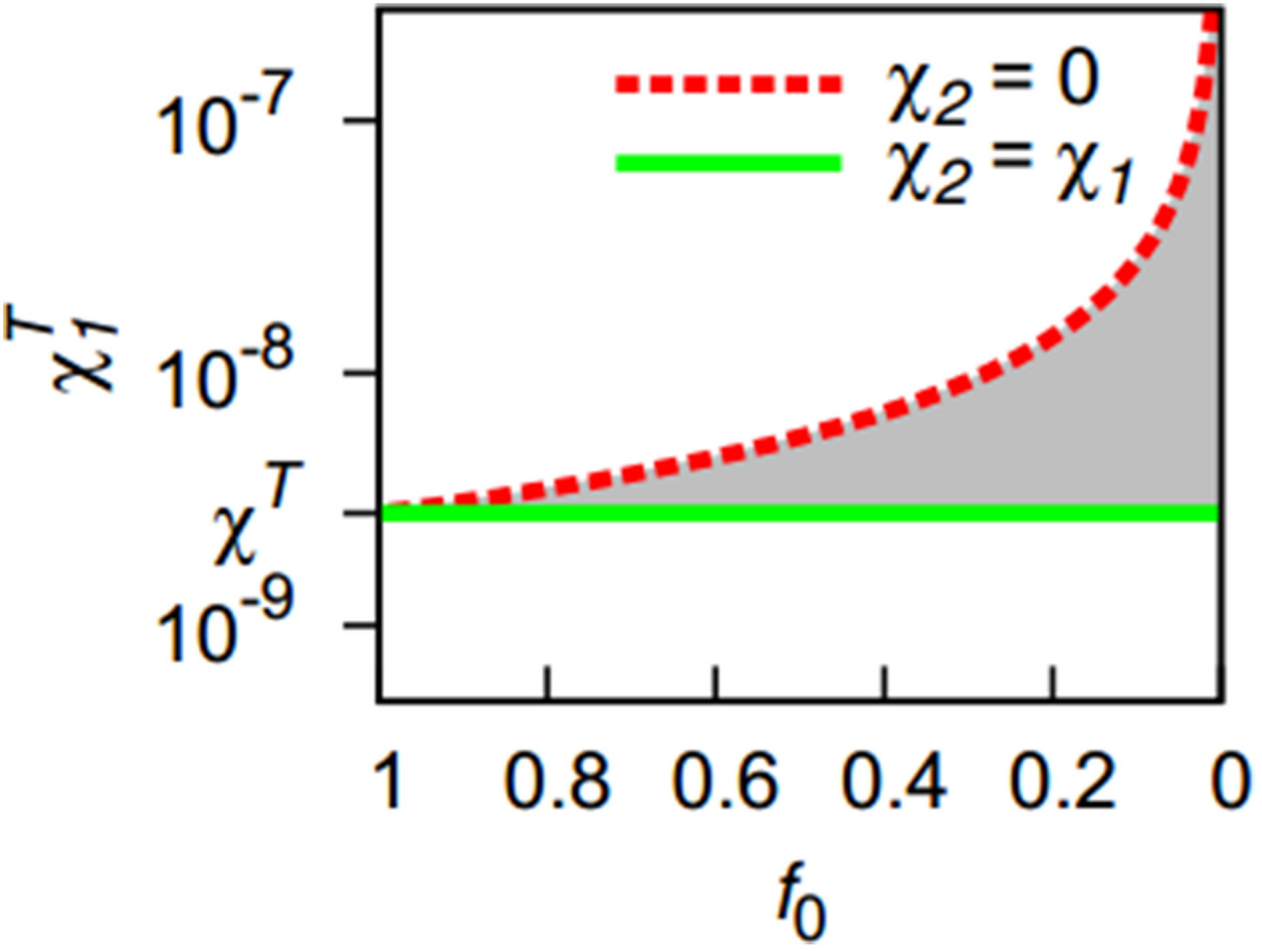
**The presence of a second, less aggregating bacterial strain (*f*_0_ < 1) increases the threshold for pattern formation with respect to the chemotactic sensitivity toward bacteria χ*^T^*_1_ of the first strain**. Lines indicate the extreme cases that the second strain has either a zero chemotactic sensitivity to bacteria (χ_2_ = 0) or shares the sensitivity of the first strain (χ_2_ = χ_1_). Except for the first extreme case, an increasing share of the less-aggregating strain (smaller *f*_0_-values) requires a stronger chemotactic sensitivity of the more aggregating strain for the onset of pattern formation. The threshold is located in the gray area for 0 < χ_2_ < χ_1_.

If the second strain has a chemotactic sensitivity between these two extreme cases, the actual threshold will be somewhere between the thresholds defined by Equations (7) and (9) (shaded area in Figure 1). To obtain an estimate, we use the ratio of the chemotactic sensitivities as a weight applied to the two extreme cases, yielding:

(10) $$\chi_{1}^{T}=\left(1-\frac{\chi_{2}}{\chi_{1}}\right)\cdot \frac{D}{f_{0}\cdot p^{*}}+\frac{\chi_{2}}{\chi_{1}}\cdot \frac{D}{p^{*}}.$$

By setting χ*^T^*_1_ to χ_1_ and solving for χ_2_, this equation can be used to provide an approximate delineation of regions in parameter space spanned by χ_1_ and χ_2_ for which homogeneous bacterial distributions and for which spatial patterns can be expected for a given initial community composition *f*_0_.

### Scenario selection and analysis

The model is always initialized with homogeneous distributions of bacterial cells and substrate, with cell numbers and substrate concentration given by the steady state of the growth model. Assuming the average cell mass to be *X_avg_* = 3/4 *X_max_*, the steady state values are p^*^ = X^*^/*X_avg_* = 2.02 × 10^−10^ g/*X_avg_* = 1856 cells and S^*^ = 3.08 × 10^−5^ g/l. Unless stated otherwise, the bacterial community is initially consisting of both strains with equal share (*f*_0_ = 0.5). We cover all three cases that both, neither, or only one strain features a chemotactic sensitivity to bacteria above the threshold for pattern formation in monocultures. In the last case, pattern formation might also depend on the initial community composition, as Equation (9) indicates. For these scenarios, additional simulation runs with *f*_0_ = 0.3 and *f*_0_ = 0.7 were performed.

All simulations covered a simulated time span of 2 weeks, which was sufficient for all scenarios except for two to settle in a stable final state. For the remaining two, the final state could be assessed reliably after an additional week of simulation time. To characterize the spatial distribution of bacterial biomass within the simulation domain, we compute the standard deviation of total cell numbers across all locations at a given time point. Low numbers will indicate homogeneous distributions where all locations share similar cell counts, while high numbers indicate heterogeneous distribution patterns. To inspect spatio-temporal patterns in detail, selected scenarios were additionally run using a larger simulation domain. All figures were created using the freely available Gnuplot plotting program, Version 4.6.

## Results

### Three spatio-temporal behaviors: homogeneous distributions, transient patterns, and permanent patterns

**Figure 2:**
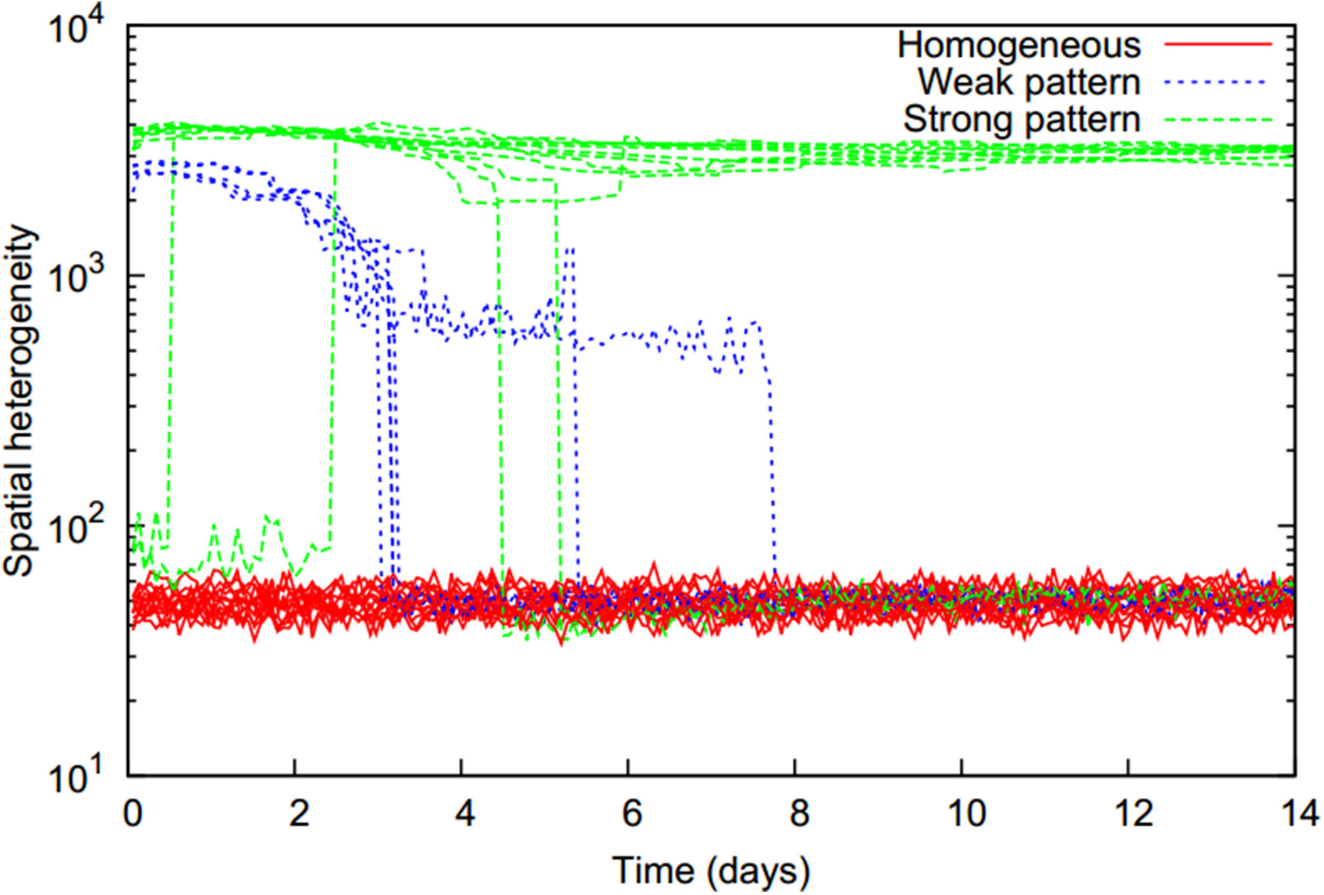
**Temporal evolution of spatial heterogeneity of the biomass distribution (computed as the standard deviation of total cell numbers over all locations, on a logarithmic scale) for all 29 simulated two-strain scenarios with initially homogeneously and equally distributed strains (*f*_0_ = 0.5) which only differed in their chemotactic preference**. Three distinct trajectories can be distinguished (colors) and refer to different pattern formation behaviors.

All simulated scenarios could be classified into three basic behaviors according to the temporal evolution of the bacterial distribution heterogeneity (Figure 2). Starting from a homogeneous state, bacterial distributions either remained homogeneous (denoted as “homogeneous”), or patterns emerged at an early time point with two different levels of heterogeneity. In lower heterogeneity scenarios (denoted as “weak pattern”), patterns abruptly dissolved at a later time point resulting in a final homogeneous bacterial distribution. Scenarios with a high level of heterogeneity (denoted as “strong pattern”) were found to typically remain in this heterogeneous state, although in a few cases a switch back to a homogenous state occurred later on.

Whenever the bacterial distribution becomes inhomogeneous, a drop in substrate consumption rates occurred, which was more severe for strong patterns (Supplementary Figure 1). This behavior has been observed before and can be explained by bacteria being drawn to locations of high bacterial densities and vacating locations where substrate is available, but can no longer be consumed (Centler et al., 2011).

**Figure 3:**
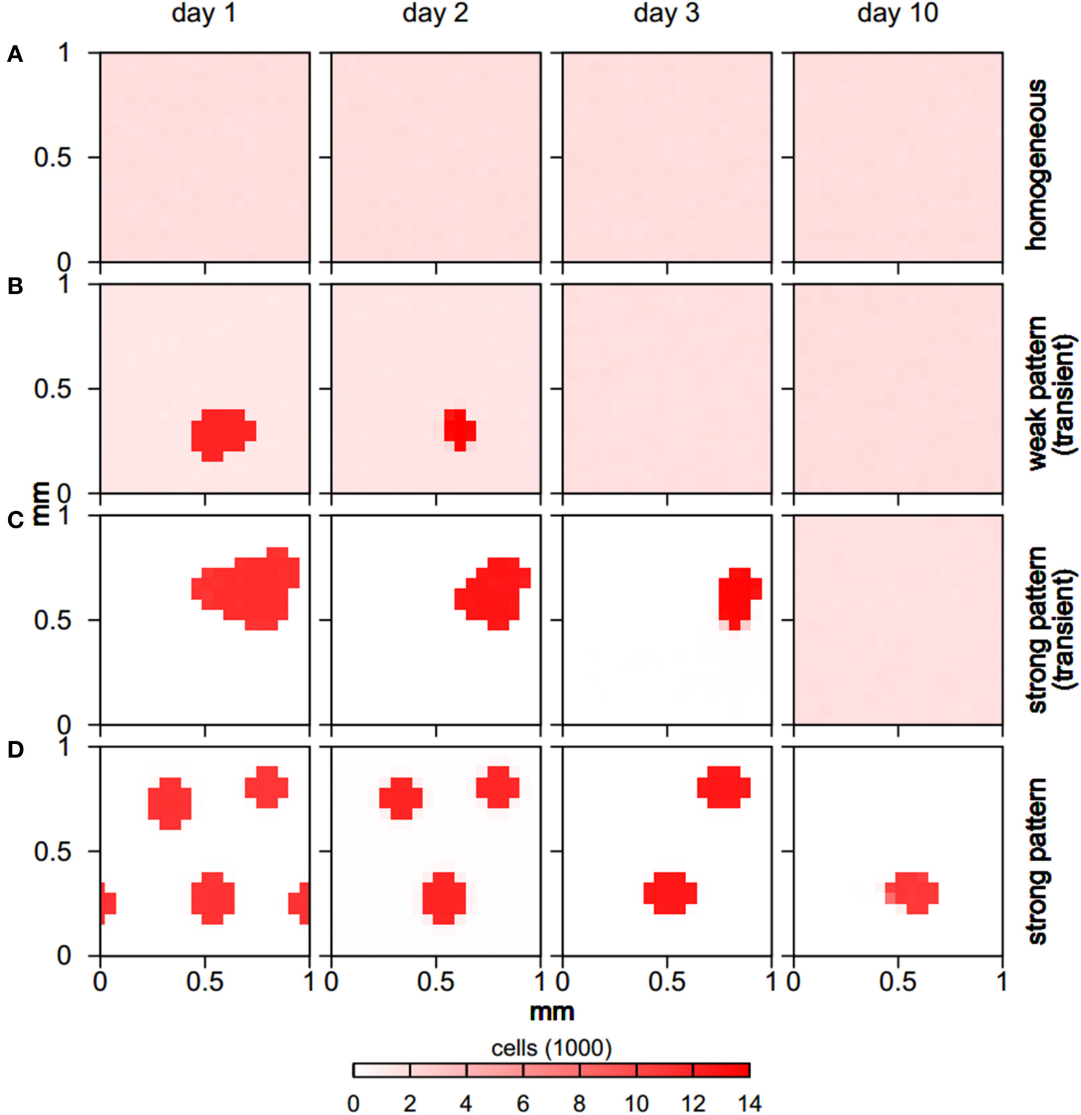
**Spatio-temporal evolution of bacterial distribution for different behaviors (rows), showing typical examples**. While the full domain is populated by microbial cells for homogeneous **(A)** and weak pattern runs **(B)**, all cells are absorbed in aggregates for strong pattern runs **(C,D)**. Aggregates either shrink and dissolve **(B,C)** leading to homogeneous final states, or undergo rearrangements but do not vanish completely **(D)**.

The explicit spatio-temporal bacterial distribution patterns also varied significantly for the distinct behaviors (Figure 3). While homogeneous runs retained a homogeneous distribution of bacterial cells throughout the simulation, for weak pattern runs bacterial aggregates formed early but then slowly shrank and disappeared over a time span of usually 3 days, with some weak patterns lasting for up to a week (Figure 2). In both, homogeneous and weak pattern scenarios, bacterial cells were populating the full simulation domain. In contrast to this, in strong pattern scenarios all cells were absorbed in aggregates. Typically, aggregate assemblages underwent rearrangements in which aggregates fused with others. In few cases, aggregates also shrank and disappeared over time as in the case of weak pattern runs. All homogeneous and transient pattern runs resulted in a final homogeneous state in which the total biomass concentration reached the steady state levels given by the growth model.

**Figure 4:**
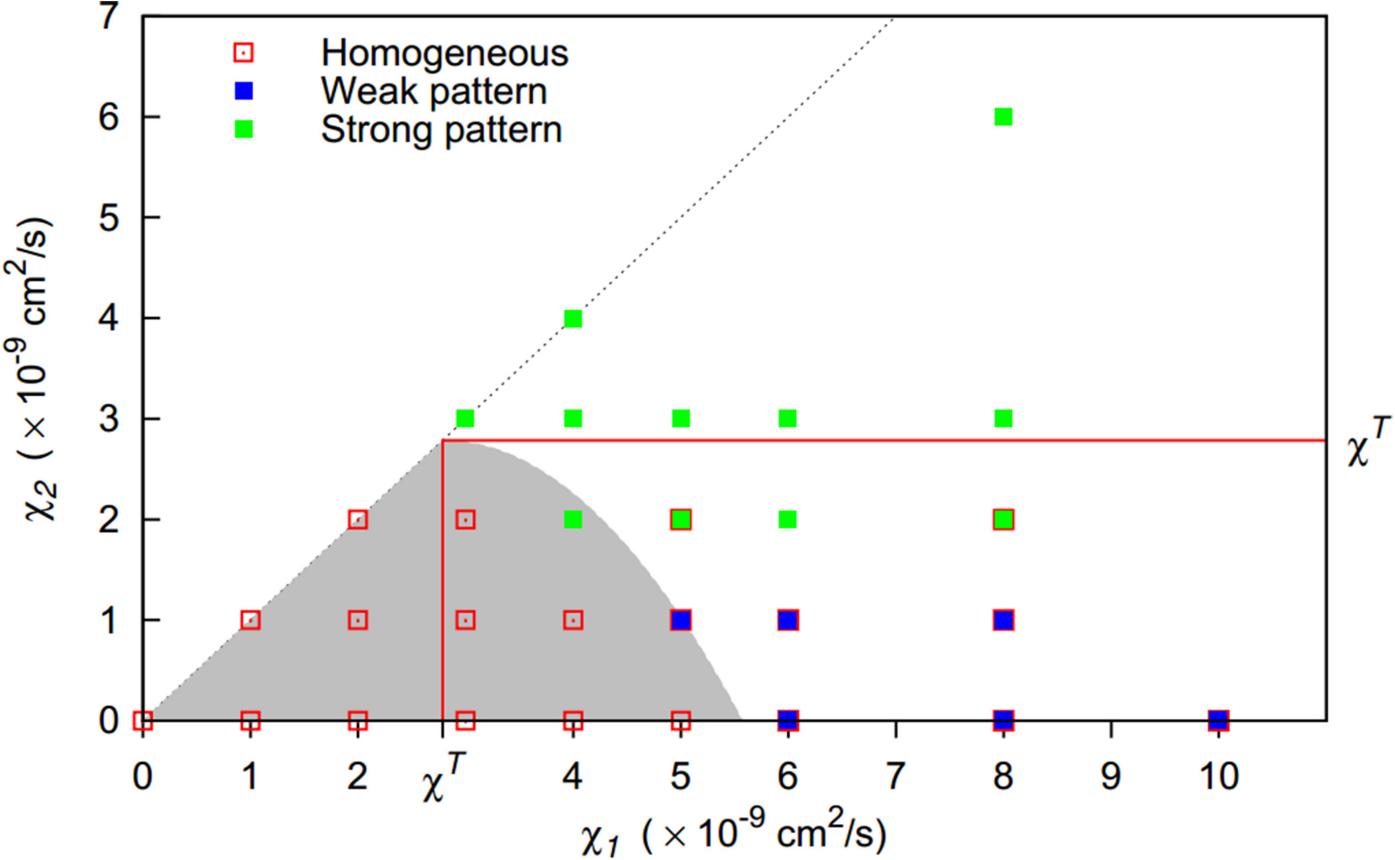
**Pattern formation behavior for two-strain communities of strains differing in their chemotactic preference (given by their chemotactic sensitivities to bacteria χ_1_, χ_2_). Initially, both populations are homogeneously initialized in the simulation domain with equal share (*f*_0_ = 0.5)**. For the gray shaded area, homogeneous bacterial distributions are expected (Equation 10). For solid symbols, red borders indicate that patterns dissolve at some time point, resulting in a final homogeneous state. Red lines indicate pattern formation thresholds in monocultures.

The three distinct behaviors could be clearly matched to regions in the parameter space spanned by the chemotactic preferences of the first and the second strain (Figure 4). If both chemotactic sensitivities to bacteria were below the pattern formation threshold in monocultures χ*^T^*, homogeneous bacterial distributions resulted. As expected, this region of homogeneous behavior extends beyond this threshold, with Equation (10) providing a reasonable estimate. For higher chemotactic sensitivities to bacteria, inhomogeneous bacterial distribution patterns emerged and the second strain's chemotactic sensitivity was decisive for the resulting behavior. For low sensitivities of up to 1 × 10^−9^ cm^2^/s, weak patterns emerged, while strong patterns emerged for higher sensitivities. If the second strain's chemotactic sensitivity was only slightly above the threshold separating weak and strong pattern behavior, strong patterns occasionally dissolved over time resulting in a final homogenous bacterial distribution. When the second strain's chemotactic sensitivity to bacteria exceeded the pattern formation threshold in monocultures, strong patterns were found to be stable.

### Competitive advantage of the less aggregating strain

**Figure 5:**
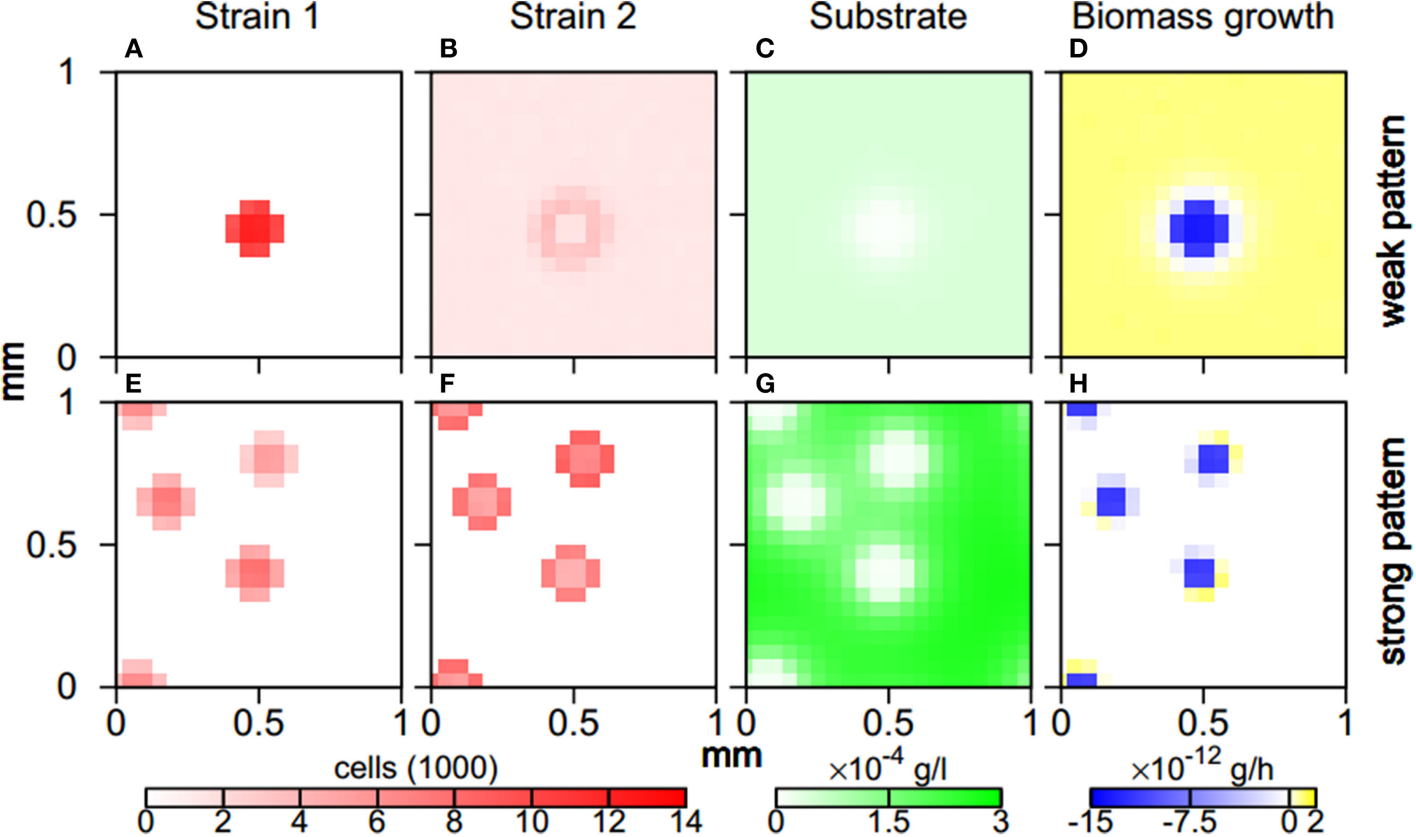
**Aggregate architecture for weak (A–D) and strong pattern behavior (E–H)**. Spatial distribution of cells of the more aggregating first **(A,E)** and the less aggregating second strain **(B,F)**, spatial distribution of substrate concentration **(C,G)**, and spatial distribution of rate of change of total biomass **(D,H)** after 2 days of simulated time. Parameter values were χ_1_ = 8 × 10^−9^ cm^2^/s and χ_2_ = 1 × 10^−9^ cm^2^/s for weak pattern behavior and χ_1_ = 8 × 10^−9^ cm^2^/s and χ_2_ = 2 × 10^−9^ cm^2^/s for strong pattern behavior.

When aggregates form during weak and strong pattern behavior, the two microbial strains segregate and populate different zones. The more aggregating first strain is solely present in aggregates. For weak pattern behavior, the second strain is present throughout the simulation domain and features a peak along aggregate fringes (Figure 5). For strong patterns, the second strain is also absorbed by aggregates where it mainly populates the fringe. Substrate levels in unpopulated regions almost reach the inflow concentration *S_in_*(Figure 5). In aggregate cores, substrate concentrations feature local minima, leading to more substrate input at the cores due to higher release rates (Equation 2), and a higher diffusive flux. Even this increased substrate input, however, cannot support the high biomass concentrations within the aggregates. Hence, for weak patterns bacterial biomass decreases within aggregates while it increases outside aggregates. For strong patterns, biomass decay is restricted to the aggregate core, while growth occurs along the aggregate fringe where substrate concentrations are sufficiently high (Figure 5).

**Figure 6:**
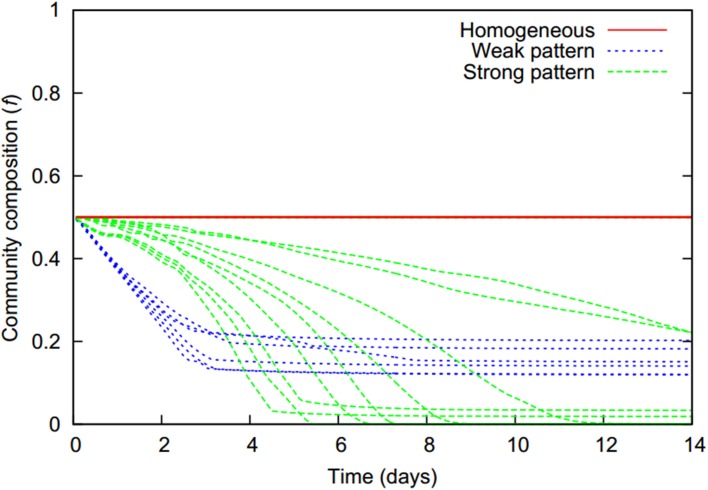
**The temporal evolution of the community composition (*f*, denoting the fraction of the more aggregating first strain) is characteristic for the different pattern formation behaviors (colors)**.

As zones of biomass decay are predominantly populated by the first strain while the second strain mainly populates the productive zones, a continuous shift of overall community composition toward the second strain occurs over time whenever microbial aggregates are present (Figure 6). As cells are starving in aggregates, respectively aggregate cores, they shrink and finally die resulting in a loss of biomass. The freed space is, however, quickly re-occupied by cells which immigrate from the productive zones being drawn to high cell densities. This balance between decay of biomass in starvation zones and the replenishing bacterial influx from the productive surroundings allows the biomass concentration in aggregate cores to remain at a constant high level, even if aggregates shrink in size as in the weak pattern scenarios. The bacterial flux toward aggregate cores leads to a slow replacement of cells of the first by cells of the second strain. In the weak pattern scenarios, at some point the number of cells of the more aggregating first strain is no longer large enough to maintain the integrity of the aggregate and it starts to dissolve, giving way to a homogeneous distribution of both cell types. This occurs typically at approximately 3 days and halts the ongoing shift in overall community composition (Figure 6).

In strong pattern scenarios the second strain's chemotactic sensitivity to bacteria is above, or close to the pattern formation threshold in monocultures. Hence aggregates do not disintegrate once they are dominated by the second strain; the first strain simply becomes fully replaced. This strain replacement without aggregate dissolution was even observed in two cases in which the chemotactic sensitivity of the second strain was below the pattern formation threshold in monocultures. This usually led to aggregate dissolution and a final homogeneous bacterial distribution in which the fraction of the first strain was, however, very small in comparison to the homogeneous distributions which unfolded in the weak pattern scenarios (Figure 6).

**Figure 7:**
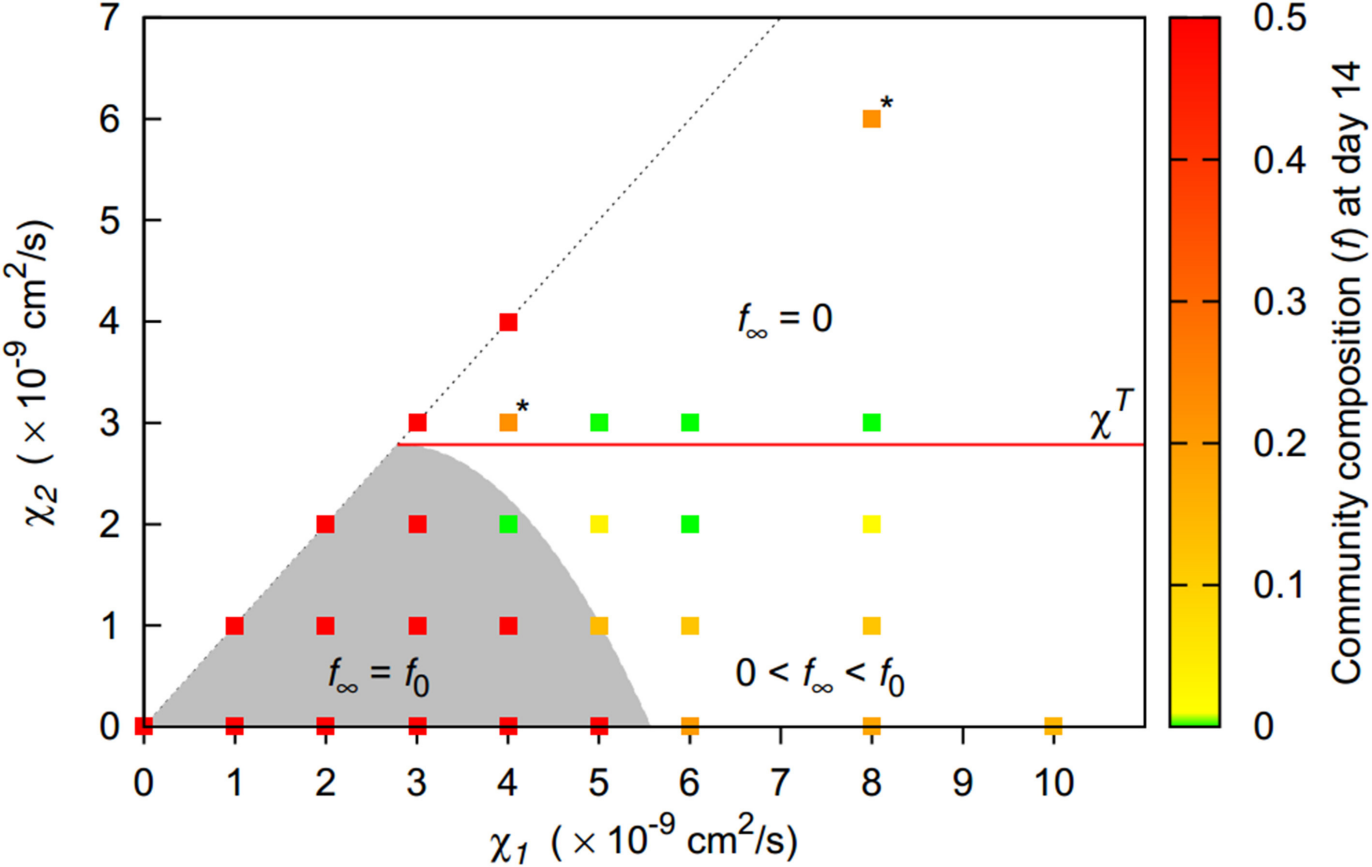
**Final community composition (*f*, denoting the fraction of the more aggregating first strain, recorded at day 14) for two-strain communities of strains differing in their chemotactic preference**. Long term coexistence with the initial community composition *f*_0_ is possible for homogenous behavior (shaded area, *f*_∞_ = *f*_0_) or if strains have identical chemotactic preferences (1:1 line). Beyond homogeneous behavior, coexistence with a dominating second strain is possible for low chemotactic sensitivities of the second strain (0 < *f*_∞_ < *f*_0_). For high chemotactic sensitivities, the first strain becomes extinct (*f*_∞_ = 0). For scenarios marked with “^*^,” *f* continued to decrease after 14 days.

Overall, the less aggregating strain had a competitive advantage whenever patterns emerged. Long term coexistence was possible whenever the second strain's chemotactic sensitivity to bacteria was low (Figure 7). For homogenous behavior, the initial community composition was maintained, while for weak patterns, a shift toward the second strain occurred. If the second strain had a chemotactic sensitivity to bacteria above the pattern formation threshold in monocultures, the second strain was even able to fully replace the first strain.

### Impact of initial community composition

Additional simulation runs with an initial dominance of the less aggregating second strain (*f*_0_ = 0.3) and a dominance of the more aggregating first strain (*f*_0_ = 0.7) confirmed that the initial community composition influences the appearance of transient patterns (Supplementary Figure 2). When the more aggregating strain was in the minority, its chemotactic sensitivity to bacteria required higher values to enable pattern formation. When it dominated the initial community composition, a lower sensitivity was sufficient for the onset of pattern formation. The theoretical expectation for homogeneous behavior given by Equation (10) was found to match the numerical simulation results reasonably well (Supplementary Figure 2).

## Discussion

In this study we considered the effect of differences in the motility phenotype on microbial competition in a simulated two-strain community populating a homogeneous environment. Strains shared identical growth characteristics and overall motility and only differed in their chemotactic preference: compared to a reference strain, a strain could be more attracted to self-excreted compounds, making it more likely to form aggregates, at the expense of sensitivity toward substrate, or vice versa. When defining a selection of strains with varying chemotactic preferences in this way and letting pairs of strains compete, individual-based simulation runs indicated that only two long term behaviors were possible: either homogeneous bacterial distribution patterns emerged with both strains coexisting, or bacterial aggregates formed and the less aggregating strain outcompeted the more aggregating strain. While absent in monocultures, transient patterns appeared in two-strain communities as a novel qualitative behavior. When aggregates were present, the less aggregating strain had a competitive advantage as it mainly populated productive zones while the more aggregating strain was restricted to zones characterized by starvation conditions. Consequently, the total community composition continuously shifted toward the less aggregating strain until either patterns vanished or the more aggregating strain became extinct. Hence, the existence of patterns, even if only transient in nature, had a strong influence on the final community composition.

For monocultures, a threshold for the chemotactic sensitivity to bacteria had been identified before above which spatial patterns emerged (Centler et al., 2011). Here, we find as expected that permanent patterns in two-strain communities emerged if both strains had chemotactic sensitivity values above this threshold, while homogeneous distributions emerged if both sensitivities were below the threshold. If one strain featured a value above the threshold while the other below, the formation of patterns was found to also depend on the initial community composition. The influence of both, the initial community composition on pattern formation, and of pattern formation on the final community composition highlight the importance of the history of a given system. An observed homogeneous bacterial distribution can be both, the result of a stable community composition of strains that do not form patterns or the result of a community shift during transient pattern formation.

We considered fast growing and very motile strains in our simulations in which transient patterns were present for up to a week and community shifts typically occurred within up to 2 weeks. For soil systems for which growth and motility can be smaller by orders of magnitudes, this transient behavior is expected to also cover equivalently longer time spans. The pattern formation behavior itself, however, is not expected to be much affected if we can assume that slower growing strains also invest less energy in random motility. In this case the ratio *D*/p^*^ remains similar, and with it the pattern formation threshold according to Equation (10).

### Pattern formation in the presence of a competing strain

The pattern formation behavior of strains in monoculture was found to be modulated by the presence of a competing strain. Both cases that pattern formation was suppressed or promoted by a second strain were observed. For a strain that forms patterns in monoculture, characterized by having a chemotactic sensitivity above the pattern formation threshold in monocultures, the presence of a second strain having a sensitivity below this threshold led, besides two exceptions, to homogeneous bacterial distribution patterns in the long term in which both strains coexisted. Hence, a strain typically forming patterns in monoculture was not able to do so in the presence of the second strain. In these runs, either the bacterial distribution remained homogeneous throughout the simulation, or transient patterns occurred, depending on the initial community composition.

The two exceptions showed the opposite behavior, that the presence of a second strain can help a strain that does not form patterns in monocultures to maintain patterns. In two scenarios at the border between week and strong pattern behavior with χ_1_ = 4 × 10^−9^ cm^2^/s or χ_1_ = 6 × 10^−9^ cm^2^/s and χ_2_ = 2 × 10^−9^ cm^2^/s, strong pattern emerged in which the first strain was finally fully replaced by the second strain. However, contrary to the fact that the second strain was not forming patterns in monocultures, here it was found able to maintain a pattern, which had been initiated by the first, more aggregating strain. While aggregates appeared to be stable over the simulated time span, random events might trigger their dissolution. No clear trend could be identified regarding the appearance of homogeneous distributions or patterns formed by non-pattern forming strains along the border between weak and strong pattern behavior. Both, the stability of such aggregates and the conditions under which they can arise need to be assessed in more detail.

The pattern formation condition assumes an initial homogeneous bacterial distribution. Here, the first, more aggregating strain led to bacterial gradients which allowed the second strain to form aggregates. An additional aggregate dissolution condition is required to assess whether a strain, starting from an aggregated distribution, is able to maintain it or not. Interestingly, stochastic events are crucial for both the formation of patterns in the model from homogeneous distributions and the dissolution of patterns maintained by non-pattern forming strains. In the former case, starting from homogeneous distributions at steady state, random fluctuation are required to create gradients which subsequently are reinforced, resulting in spatial patterns. In the latter case, a random event in which many cells detach from the aggregate and leave aggregate cell numbers below a critical threshold might trigger its dissolution.

### Coexistence and aggregate dynamics

The possibility of coexistence of two competing species has been assessed considering a trade-off between growth and chemotactic response to the substrate (Kelly et al., 1988), and in the absence of chemotaxis under the assumption of identical growth and selecting random motility as the only difference between competing species (Weisman and Kessler, 2013). In both cases, heterogeneous environments were considered. For the homogeneous environments considered in the present study, coexistence is rather expected as both strains share the same growth phenotype. Nevertheless, transient patterns were shown to shift the community composition, and for permanent patterns one strain even became extinct. If both strains are forming patterns in monocultures, we would have expected to also see coexistence in the case of permanent patterns, with some aggregates consisting only of cells of the first strain, while others of the second. The homogenous initial distribution of both strains, however, seems to prevent such an outcome. The observation of strain extinction might hence solely depend on the selected initial conditions and might not be observed in more realistic scenarios. Indeed, if considering an unmixed initial distribution of two pattern forming strains, coexistence would be observed on the large scale with individual aggregates being formed either by the first or the second strain. For the other cases, an unmixed initial state would not lead to further insights: if both strains are not forming pattern, they would simply mix over time. In case only one strain is pattern forming, the other would mix into the aggregates formed by the first strain and start to cause their disintegration, as has been observed in our simulations.

Whenever aggregates formed, the less aggregating strain populated the productive fringe zone while the more aggregating population was restricted to the aggregate core, where starving conditions prevailed. This architecture of a productive fringe zone surrounding a zone characterized by high bacterial density but low productivity resembles the typical situation in a biofilm (Serra et al., 2013). While cells at the fringe have access to ambient substrate concentrations, growth in the biofilm's interior is limited due to the diffusive flux of the leftover substrate from the fringe. In our model, cells in aggregate cores shrank and died due to the starvation conditions. This allowed cells in the productive fringe zone to invade the core as they were chemotactically attracted to the high bacterial density core zone. For permanent patterns, this process continued until the second strain fully populated both the core and the fringe zone, completely displacing the first strain. Despite the model's simplified representation of a microbial system, such a phenomenological behavior has been observed in an experimental system, although being driven by a different mechanism. Planktonic swimming bacteria were shown to infiltrate biofilm structures by burrowing deep tunnels (Houry et al., 2012). These tunnels increased mixing within the biofilm and exposed it to ambient concentrations of both substrate and potentially toxic substances. For a swimming bacterium producing an antimicrobial compound, the eradication of the biofilm and the successive colonization by the swimming bacterium was observed.

### Optimal strategy and evolutionary implications

In scenarios where transient or permanent patterns occurred, the less aggregating strain always increased its share on the total population. From this observation the optimal strategy can be easily deduced: be equally or less aggregating than the competing strain. This strict rule can be relaxed for scenarios where homogeneous bacterial distributions were maintained throughout the simulation. In these cases, the initial community composition was maintained, indicating no competitive advantages, although chemotactic preferences varied for both strains. If one strain does not form patterns in monoculture, the competing strain can feature higher chemotactic sensitivities of at least up to the pattern formation threshold in monocultures, and depending on the initial community composition even beyond. As long as no patterns emerge, chemotactic preference has no influence on competitiveness.

These findings have implications for the evolution of the ability to form aggregates in our model. If we assume that the chemotactic preference is the only trait affected by evolutionary forces, we first consider the consequences for a pattern forming strain. If a subpopulation evolves that features a higher chemotactic sensitivity to bacteria, it will be outcompeted by the ancestor population. If however, a subpopulation with a lower chemotactic sensitivity evolves, it will replace the ancestor population. This process repeats itself until the new subpopulation has a chemotactic sensitivity below the threshold for pattern formation in monocultures. In this case, both populations coexist in a homogeneous distribution. As a result, a pattern forming strain will always tend to lose this ability in our model. But can this ability evolve from a strain which is not forming patterns in the first place? Starting from a monoculture having a chemotactic sensitivity below the pattern formation threshold, slightly higher or lower sensitivities might occur by random genetic drift, having no effect on the competitiveness of the respective cells as long as the resulting bacterial distribution remains homogeneous. As the region of homogeneous behavior extends beyond the pattern formation threshold in monocultures in the presence of a second strain, also values beyond this threshold can occur by neutral evolution. If cells having such chemotactic sensitivities become spatially separated from the community, for example by advective transport or random motility, they as a monoculture will then have the ability to form aggregates.

While the ability to form aggregates can arise by chance in our model, to maintain this ability, it must provide a benefit. This is, however, not the case in our model and on the contrary, aggregating strains become fixed to their aggregates, while non-aggregating strains remain free to explore the space outside aggregates, providing an ecological advantage (Picioreanu et al., 2007). If an aggregated lifestyle provides a benefit, for example more effective substrate utilization or enhanced resistance to environmental stress, the evolution and maintenance of this ability will naturally become much more likely. If however, this benefit is the result of an adaptive evolutionary process requiring first individual cells to reach high densities over prolonged time periods, our model indicates how such an aggregated lifestyle might evolve. The ability to form aggregates arises by chance in a subpopulation of an ancestor population. The presence of the ancestor population, however, prevents actual aggregate formation and its associated negative effects. Only after spatial separation from the ancestor population, an aggregated lifestyle unfolds. The initial disadvantage of increased competition due to high cell densities can then be more than compensated for if a social, co-operative lifestyle evolves.

While our simplified model indicates a mechanism by which an initially disadvantageous trait can evolve, natural evolution is certainly much more complex, acting on many microbial traits simultaneously instead of the one considered here, and operates in the context of spatial heterogeneities and temporal fluctuations which were equally neglected in this study. While the model's simplifications and focus on selected aspects allow for a clear identification of principles and cause and effect, their ultimate relevance must be assessed in the context of the full complexity of the natural system.

## Conclusion and outlook

Using a model that simplifies many aspects of natural environments we analyzed the impact of motility characteristics on competition and pattern formation in two-strain microbial communities. Results indicated that motility aspects, here the chemotactic preference which was varied from strain to strain, have a major impact on microbial distribution patterns and on the temporal evolution of community compositions. Simulations showed how the behavior of strains in monoculture was modulated by the presence of a second strain, indicating that interactions beyond direct substrate competition were influencing the community fate. These gave rise to transient patterns as a novel qualitative behavior which was not observed in monocultures. Hence, mixed systems can display a more complex dynamic repertoire which might not be predictable from results obtained for monocultures.

While for monocultures, the ability to form patterns from initially homogeneous bacterial distributions was only depending on the random bacterial motility and the steady state bacterial density (Equation 7), for mixed two-strain communities this ability additionally depended not only on the other strain's chemotactic sensitivity to bacteria, but also on the initial community composition (Equation 10). With the latter potentially giving rise to transient patterns, these results highlight the importance of the history of a community when analyzing a given bacterial distribution. An observed stable coexistence could both be the result of an initially stable or unstable community, with the latter having undergone a shift in community composition. Additionally, in two cases the transient presence of a second strain was required to generate the final bacterial distribution. In these cases, the observed final distribution could not have been explained without knowledge on the community's history, as no trace of the transient presence of the second strain was left.

Considering fast growing bacterial strains of high motility in our model, steady states were usually reached in 1–2 weeks. In natural environments, where growth and motility are expected to be orders of magnitudes smaller, relaxation times will accordingly be longer. Comparing these with typical frequencies of disturbances such as rain events, natural systems might often never reach a steady state. When experimentally following the fate of a community in natural environments, our findings indicate that taking samples at different times over long time periods is mandatory to capture the potentially slow dynamics of these systems.

While competition for substrate was the only direct species interaction considered in the model, with both species featuring identical growth phenotypes, the model is flexible enough to accommodate other interactions, for example the production and effect of antibiotics. Along these lines, the importance of random motility for biodiversity in the presence of cyclic interactions has been assessed, albeit in the absence of chemotaxis (Adamson and Morozov, 2012). Abiotic heterogeneity can also be considered, for example if substrate is only supplied at certain locations in the domain, leading to more pronounced substrate gradients. Such a scenario would more resemble the situation of preferential flow paths in the subsurface. Abiotic heterogeneities have been considered in the context of competing growth strategies (Stolpovsky et al., 2012), and their interplay with patterns formed by chemotactic bacteria has been explored before (Gharasoo et al., 2014). Going beyond static heterogeneities, the location of substrate supply could also be varied over time. The departure from static environmental conditions would dramatically change the relevance of motility aspects and likely has profound consequences for microbial competition. Finally, results obtained here led to hypotheses regarding the evolution of microbial behavior. By incorporating random variations in bacterial parameters during cell division, these are readily testable and can be refined in follow-up simulation studies. The chosen individual-based modeling approach is an excellent opportunity to investigate questions relating to microbial evolution, providing hypotheses which are subsequently testable in laboratory experiments.

## Author contributions

Florian Centler and Martin Thullner conceived the study, Florian Centler implemented the model, performed the simulations and analyzed the data, and both wrote the manuscript.

### Conflict of interest statement

The authors declare that the research was conducted in the absence of any commercial or financial relationships that could be construed as a potential conflict of interest.
