# Supplementary material for: Chemotactic preferences govern competition and pattern formation in simulated two-strain microbial communities
Source: Front Microbiol. 2015 Feb 2;6:40. doi: 10.3389/fmicb.2015.00040 (PMC4313714; doi:10.3389/fmicb.2015.00040)
Supplement: Supplementary file 1 [file DataSheet1.DOCX]

***Supplementary Material***

**Chemotactic preferences govern competition and pattern formation in simulated two-strain microbial communities**

**Florian Centler*, Martin Thullner**

Department of Environmental Microbiology, UFZ – Helmholtz Centre for Environmental Research, Leipzig, Germany

*** Correspondence:** Dr. Florian Centler, Department of Environmental Microbiology, UFZ – Helmholtz Centre for Environmental Research, Permoserstr. 15, 04318 Leipzig, Germany.

florian.centler@ufz.de

1. **Supplementary Figures**

Supplementary Figure 1 Total substrate consumption decreases as spatial patterns emerge in the bacterial distribution. For transient weak pattern scenarios this decrease is temporary and small compared to the decrease in strong pattern scenarios.

Supplementary Figure 2 Influence of initial community composition on pattern formation behavior: if the initial composition is dominated by the second, less aggregating strain with *f*_0_ = 0.3 more scenarios show homogeneous behavior (A) than if the more aggregating strain dominates the initial community composition with *f*_0_ = 0.7 (B). The gray shaded area indicates the expectation for homogeneous behavior (Equation 10) and for comparison, the dashed line indicates the border for *f*_0_ = 0.5. For solid symbols, red borders indicate that patterns dissolve at some time point, resulting in a final homogeneous state. Red lines indicate pattern formation thresholds for monocultures.
