# Supplementary figures and images for: Chemotactic preferences govern competition and pattern formation in simulated two-strain microbial communities
Source: Front Microbiol. 2015 Feb 2;6:40. doi: 10.3389/fmicb.2015.00040 (PMC4313714; doi:10.3389/fmicb.2015.00040)

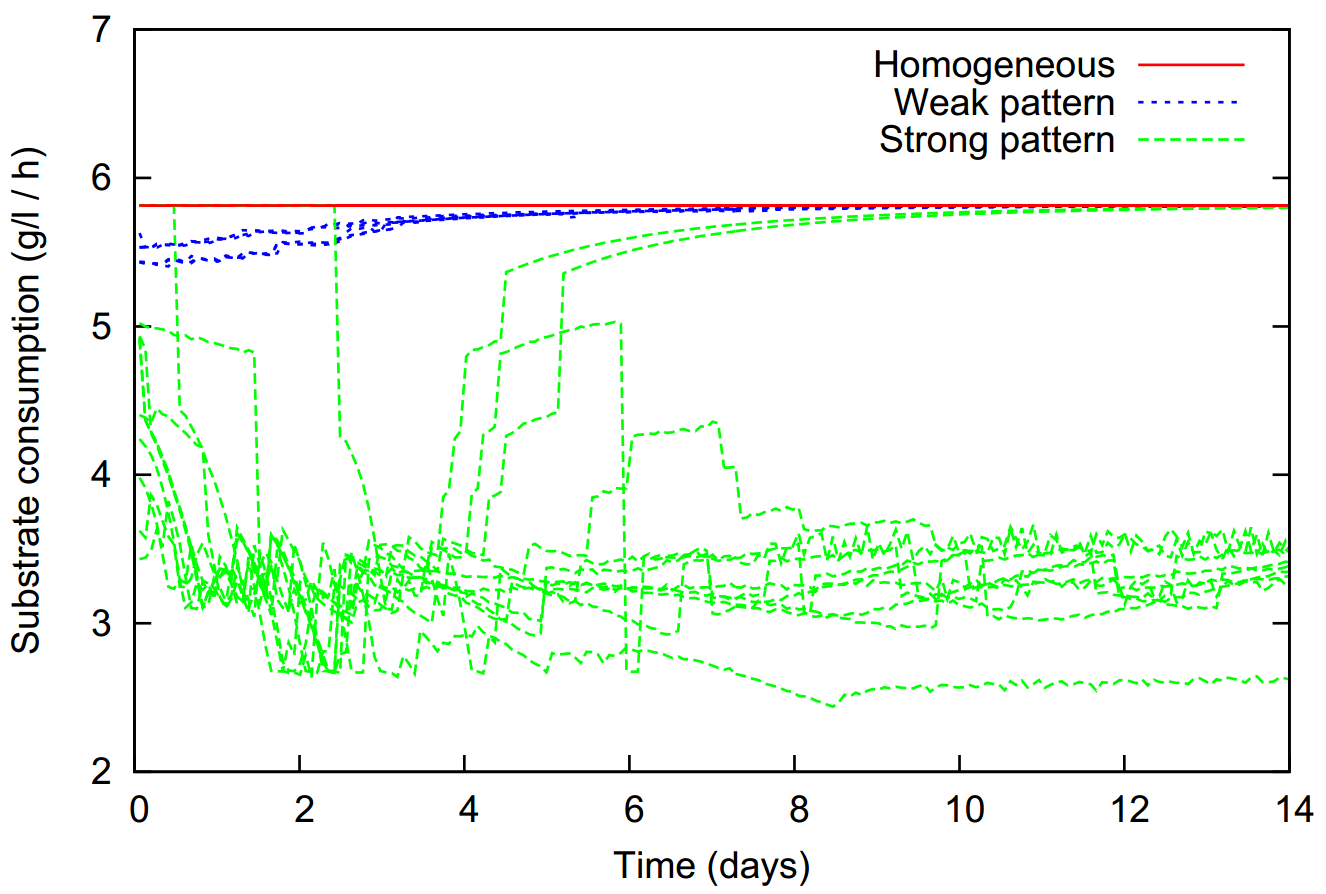

Supplement: Supplementary file 2 [file Image1.TIF]

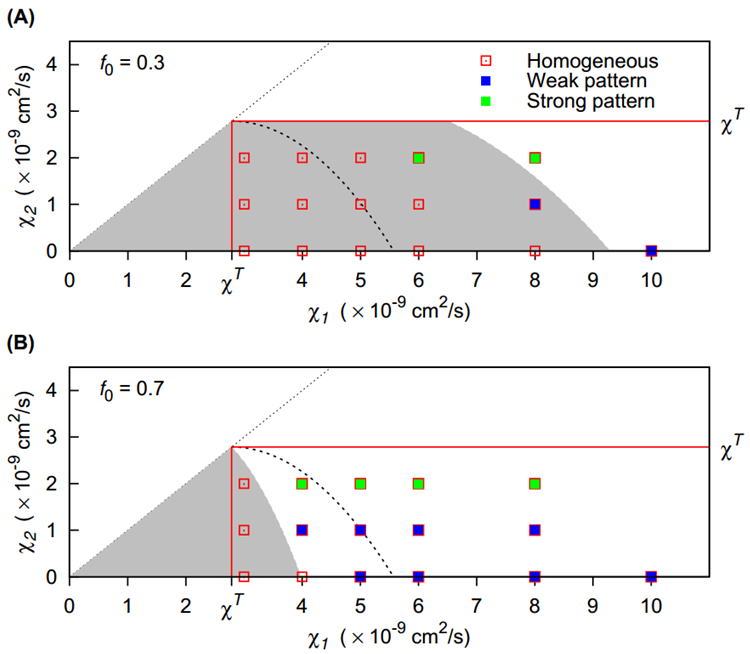

Supplement: Supplementary file 3 [file Image2.TIF]
